# Reconstructing Bone with Natural Bone Graft: A Review of In Vivo Studies in Bone Defect Animal Model

**DOI:** 10.3390/nano8120999

**Published:** 2018-12-03

**Authors:** Mengying Liu, Yonggang Lv

**Affiliations:** 1 Key Laboratory of Biorheological Science and Technology (Chongqing University), Ministry of Education, Bioengineering College, Chongqing University, Chongqing 400044, China; Liumeng0391@sina.com; 2 Mechanobiology and Regenerative Medicine Laboratory, Bioengineering College, Chongqing University, Chongqing 400044, China

**Keywords:** natural bone graft, decellularized bone matrix scaffold, demineralized bone matrix scaffold, animal defect model, bone reconstruction

## Abstract

Bone defects caused by fracture, disease or congenital defect remains a medically important problem to be solved. Bone tissue engineering (BTE) is a promising approach by providing scaffolds to guide and support the treatment of bone defects. However, the autologous bone graft has many defects such as limited sources and long surgical procedures. Therefore, xenograft bone graft is considered as one of the best substitutions and has been effectively used in clinical practice. Due to better preserved natural bone structure, suitable mechanical properties, low immunogenicity, good osteoinductivity and osteoconductivity in natural bone graft, decellularized and demineralized bone matrix (DBM) scaffolds were selected and discussed in the present review. In vivo animal models provide a complex physiological environment for understanding and evaluating material properties and provide important reference data for clinical trials. The purpose of this review is to outline the in vivo bone regeneration and remodeling capabilities of decellularized and DBM scaffolds in bone defect models to better evaluate the potential of these two types of scaffolds in BTE. Taking into account the limitations of the state-of-the-art technology, the results of the animal bone defect model also provide important information for future design of natural bone composite scaffolds.

## 1. Introduction

Bone is an organ that plays a major role in supporting and protecting. Mature bone tissue consists mainly of 20% cancellous bone and 80% compact bone [1]. Cancellous bone is composed of high-porosity trabecular bone with a porosity of 50% to 90%, and the inside of which is like a sponge, has the same branch as honeycomb [1,2]. Compact bone generally has 10% porosity. The microenvironment with high porosity and high stiffness formed by natural bone not only plays the role of mechanical support, but also facilitates the spread and adhesion of bone stromal cells on it and provides sites for the deposition of new bone matrix.

Tumor resection, pathological deformation, congenital deformation, sports injury, and infection all may cause bone defects [3,4], and even lead to osteoarthritis or other diseases, which can damage the normal musculoskeletal system of the human body. There are more than 1.5 million patients undergoing bone graft surgeries every year in the world, with market turnover of about 1.5 billion dollars [3,5]. Although great progress has been made in bone repair in recent years, real morphological and functional reconstruction of bone defects still faces enormous challenges as bone repair is a complex process. Different types of cells, signaling molecules and matrix proteins work together to repair bone defects. For decades, novel methods and technologies of bone tissue engineering (BTE) have been introduced to address the problem, including autologous bone graft, allogeneic bone graft, xenogeneic bone graft and the development of various biosynthetic grafts. Some semi-natural or fully synthetic scaffolds were also developed and applied in clinical practice. For example, the real potential for osteochondral regeneration of nanostructured collagen-hydroxyapatite (Col-HA) multilayer scaffold was assessed by Marcacci’s group [6]. A total of 100 patients affected by symptomatic chondral and osteochondral lesions were treated with the scaffolds up to 2 years. Patients affected by deep osteochondral lesions exhibited statistically significant better international knee documentation committee knee uation form (IKDC) subjective results after implantation of the Col-HA multilayer scaffold. Marcacci’s group also highlighted the safety and potential of biomimetic implants in cartilage healing and osteochondral reconstruction and provided a systematic review of preclinical and clinical evidence [7,8,9].

Due to the unique physiologic microstructure of natural bone and guidance for bone development and physiologic regeneration, an ever-increasing number of synthetic grafts are designed to mimic bone microstructure [10,11,12], such as better osteoconductive ceramics [13] and biodegradable synthetic polymers [14]. Bioceramics are classified into two types: Bio-inert ceramics (such as alumina and zirconia) and bioactive ceramics (such as hydroxyphosphite, tricalcium phosphate, and bioactive glass (calcium phosphate)). After careful design, bioceramic materials can mimic tiny pores of the human bone, and provide space for cell adhesion and growth. There are still some shortcomings in the use of bioceramic materials for bone tissue repair, such as poor mechanical conductivity, unsatisfactory physical and mechanical properties, high brittleness, poor absorption and uncontrolled and unpredictable in vivo degradability [2]. Synthetic scaffolds always lack osteoinductive signals [15]. For example, Levingstone et al. [16] demonstrated that cell-free multi-layered collagen-based scaffolds presented superior layer specific regeneration of functional osteochondral tissue in caprine joints compared with market approved synthetic polymer scaffolds. Considering the limitations of the above grafts, natural derived bone that automatically retains natural bone structure not only has similar hardness as human bone, but also has similar components, porosity and pore size. In addition, it is an ideal vehicle for tissue engineering due to its good biocompatibility and absorbability. Natural derived bone refers to cortical or cancellous bone undergone further processes to form a specific shape (e.g., cuboid and cylinder) of decellularized bone matrix, demineralized bone matrix (DBM) or other bones, is always used to fill the empty bone defects. The significant advantage of decellularized bone matrix and DBM is that they reserve the macro-structural features (e.g., geometry, pore shape and size distribution) and micro-structural features (e.g., surface roughness, nanotopography and presence of micropores) in the natural bone, which was widely reported to greatly influence the osteoinductivity of bone graft [17,18]. Acellular matrix can effectively reduce immunogenicity and avoid allergic reaction and disease transmission. After decalcification, DBM can expose more osteogenesis-related factors. Some studies have reported that natural and synthetic scaffolds can also be used to reproduce and study the biomechanical interactions between cancer cells and surrounding extracellular matrices (ECMs) and their effects on tumor phenotype and behavior, as studied by the Ibrahim group [19]. They studied the mechanical biology of cancer cell-ECM interactions via a collagen-based three-dimensional (3D) scaffold. Consequently, acellular bone and decalcified bone provide promising alternatives to develop bone graft and a foundation for regenerative efforts.

In vitro evaluation, which is often used to assess the biocompatibility, mineralization and cytotoxicity of biomaterials, is essential in estimation of material properties. Although cells and signaling factors can be artificially loaded into matrices or scaffolds, in vitro assessment cannot fully reproduce the complexities of the real environment in vivo, in particular the lack of immune or inflammatory responses [20] and a series of cascade reactions to repair bone damage in vivo. Moreover, it is easy to overestimate the cytotoxicity of materials in vitro, and most current in vitro studies were done under a two-dimensional (2D) environment [20,21]. Based on the specific limitations of the in vitro assessment described above, animal models of bone defects have been applied to the study of biomaterials and have been discussed comprehensively in some review papers [21,22].

Selecting a particular animal as a model requires considering more factors. Animal species, animal age, defect size, defect generation method, duration of study, and fixation method all affect bone repair of graft after implantation [23,24]. Most importantly, the selected animal should show similar physiological and pathological conditions to humans [20]. After controlled surgical procedures, various criteria can be used to evaluate the repair effect in a relatively short period of time [20]. Particular attention should be paid to compliance with international standards in all procedures, including the acquisition and rearing of animals. Possible bone defect models include non-load-bearing models, for example, the calvarial defect model is always applied in poorly mechanical bone scaffolds, whereas load-bearing bone defects are typically established on the femur, tibia, and so on.

The aim of this article is therefore to highlight the current focus of two kinds of natural bone scaffolds: Decellularized bone scaffold and DBM scaffold, and their respective advantages and disadvantages. In addition, the capacity of in vivo bone regeneration and reconstruction of decellularized bone scaffold and DBM scaffold in different bone defect models was reviewed to better understand and evaluate the potential applications of these two types of scaffolds in BTE.

## 2. Natural Bone Graft

### 2.1. Decellularized Bone Matrix Scaffold

**Figure 1:**
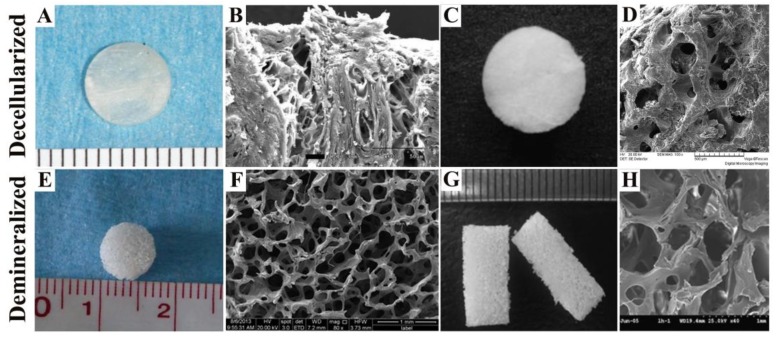
Macroscopic views and scanning electron microscope (SEM) micrographs of decellularized bone and demineralized bone. (**A**) Decellularized rat calvaria. Scale bar: 1 mm. (**B**) The porous structure of decellularized rat calvaria. Scale bar: 50 μm. (**C**) Cartilage ECM-derived scaffold from human decellularized cadaveric joint of approximately 5 mm in diameter. (**D**) The porous structure of decellularized pig cancellous bone. Scale bar: 500 μm. (**E**) Decalcified bone of pig. Scale bar: 10 mm. (**F**) The porous structure of decalcified bone from pig. Scale bar: 1 mm. (**G**) Decalcified bone of bovine. Scale bar: 10 mm. (**H**) The porous structure of decalcified bone from bovine. Scale bar: 1 mm. Reproduced with permission [29]. Copyright Dong Joon Lee et al., 2014. Reproduced with permission [30]. Copyright Elsevier B.V., 2008. Reproduced with permission [31]. Copyright American Chemical Society, 2015. Reproduced with permission [41]. Copyright Wang et al., 2014. Reproduced with permission [42]. Copyright Elsevier B.V., 2007.

Decellularized bone is the skeleton structure of natural ECM obtained by using physical methods (snap freezing, mechanical force and mechanical agitation, etc.) [25], chemical methods (alkaline solution, acid, nonionic detergents and Tritonn X-100, etc.) [26], enzymatic methods (exonucleases, endonucleases and trypsin, etc.) [27] or other methods to effectively remove the cells within the host bone tissue (Table 1) [28]. Figure 1A,C shows the macroscopic views of rat calvaria and cartilage ECM-derived 3D interconnected porous scaffold from human cadaveric joint for in vivo cartilage tissue engineering after decellularization [29,30]. The surface appearance of decellularized rat calvaria and decellurized pig femoral head of white swine is shown in Figure 1B,D [29,31]. Decellularized bone reduces or eliminates bone antigenicity, has appropriate mechanical strength and good biocompatibility, and has the same structure and composition as native bone (Table 1). Decellularized bone is widely used as a bone graft substitute because it is thought to provide a good cell microenvironment for a variety of stem cells [32]. In addition, decellularized bone has been proved to induce bone tissue repair and modulate its metabolic activity by recruiting appropriate endogenous cell subsets [18,33].

There are several key points to note when preparing acellular bone scaffolds. The first is the decellularized method, which affects the biochemical composition, tissue ultrastructure, mechanical behavior of bone scaffolds and the host response during in vivo experiments [28]. In other words, the acellular method will inevitably cause damage to the natural ECM composition and microstructure. In order to minimize such damage while maintaining maximum decellularization, continuous optimization of methods is a long-term research goal in the field of BTE. One of the main reasons that limit the clinical exploitation of decellularized bone scaffolds is the lack of a suitable sterilization method [34]. The ideal sterilization method must eliminate all the bioburden presented in the biological material while avoiding significant changes in mechanical and biological properties as these may compromise the integrity and function of the implant [34]. Current methods of sterilization include the use of heat and pressure (dry heat, moist heat), ionizing radiation (ultraviolet light, X-rays, γ-irradiation and electron beam), chemical germicides (ethylene oxide, hydrogen peroxide and peracetic acid) and some technologies based on supercritical carbon dioxide [35] and gas plasma [34]. However, these sterilization processes can damage tissue ECM components, change their biomechanical and physiological properties and cell adhesion [35,36]. For example, Somers et al. [37] found that the ultrastructure of decellularized valves subjected to gamma irradiation has been changed during in vitro experiments [38], including molecular fragmentation, degradation of protein materials and cross-linking, all of which result in significant changes in the mechanical properties of the material. Furthermore, Matuska et al. [39] showed that these unfavorable structural changes contribute to poor cell adhesion. Therefore, a new sterilization method should be designed to achieve effective sterilization without compromising the ECM’s components. In addition, differences in ECMs among different donor sources are difficult to rule out and it is difficult to completely avoid inflammation and immune responses of donor-provided bone tissue (Table 1) [40]. By purposeful and specific modification of the decellularized bone scaffold (e.g., coating or doping some functional components), these disadvantages can be ameliorated and better effects of bone reconstruction can be achieved.

### 2.2. Demineralized Bone Matrix Scaffold

DBM is a complex of collagen, non-collagen and lower concentrations of growth factors made up of a series of physicochemical treatments (multiple chemical reagents) (Table 1) [43]. Figure 1E,G shows the spongy bone of pig and the spongy bone of bovine after demineralization [34,35]. Their surface appearance is shown in Figure 1F,H [34,35]. In 1965, Urist [44] demonstrated that DBM can induce osteogenesis for the first time. Subsequently, Urist and his colleagues [45] proposed that bone morphogenetic proteins (BMPs) are the key factor of osteogenic activity. DBM has excellent biological properties, osteoinductive, osteoconductive, biodegradability, promotes bone mineralization, and accelerates bone healing [46]. As an allograft bone graft, the natural advantages (Table 1) of decalcified bone are as follows: (1) Decalcification removes the dense mineral components which wrap around BMPs and other growth factors, consequently these factors can be released smoothly and exerts osteogenic activity [47,48]; (2) DBM is one of the weakest immunogens in alternative bone graft materials after being treated with various chemical agents; (3) DBM has the natural bone composition, reticular pore structure and 3D structure. It also has good affinity and binding force, so it can be used as a natural carrier of a variety of growth factors, which was conducive to seed cell adhesion and growth. In the research of Lv’s group [49], live/dead assay of DBM scaffolds with different stiffness cultured for 3 days revealed that the cells adhered and grew well on them. After 3 weeks of culture, cell density was relatively constant in all groups, and most mesenchymal stem cells (MSCs) were viable. Furthermore, DBM provides a large surface area and internal space for ECM secretion [50].

However, there are also some disadvantages (Table 1) that limit the application and development in DBM. After being treated with various chemical agents, DBM loses most of the inorganic components, so it has low biomechanical properties. The mechanical strength of DBM is only 40% to 60% of normal bones. It can only be used as filling material and is not suitable for repairing bone defects in load bearing sites. Therefore, DBM is often combined with other stem cells, growth factors and biomaterials to effectively repair bone defects. For example, Kim et al. [51] implanted DBM combined with adipose-derived stem cells (ADSCs) into the skull defects in rats and found that DBM promoted the formation of new bone. Dallari et al. [52] combined DBM with hydroxyapatite (HA) to form nanocomposites, and found that the vacancy was completely filled after being implanted in the tibial defect for a period of time.

There are a number of factors that influence the osteogenic effects of DBM, such as species, age, gender [53], degree of decalcification, method of disinfection [54] and particle size [55]. Among them, evaluation of bone repair of DBM with different degrees of decalcification has been widely reported. Different degrees of decalcification lead to different mechanical strengths to accommodate the clinical needs of different sites of bone [56], while meeting the requirements of different stiffness matrices for endogenous cell growth. As early as the end of 20th century, Zhang et al. [57] found that decalcification affected bone-induced osteogenic activity. When the residual calcium content was 2% of the decalcified bone matrix weight, the induced new bone mass was the highest. Additionally, new bone mass was decreased when the residual calcium content was less than 1.2%. Therefore, a proper decalcification of bone matrix can become a calcified core of new bone and provide nuclei for the deposition of calcium phosphate. Moreover, studies have uncovered that the bone healing of partially DBM was significantly better than that of non-DBM and fully DBM [56]. Therefore, the study on different decalcification duration has also been the research focus of DBM scaffolds.

## 3. Summary of In Vivo Studies in Bone Defect Animal Model

### 3.1. Rat Model Studies

A total of 339 articles were retrieved from the PubMed database of National Center for Biotechnology Information (NCBI) using the keyword of “decellularized bone or demineralized bone, rat defect model.” It was found that literature on demineralized bone is 10 articles more than those on decellularized bone. At the same time, it was also found that more literature was published 10 years ago, and less high-score papers published in the past three years. Moreover, most of the literature focuses on the repair of rat calvarial defects and rat femoral defects, while other repairs such as mandibular and fibula only account for a small portion. In vivo results of different natural bone grafts implanted in different parts of the rat bone defect model are shown in Table 2.

The rat germline will affect the bone defects healing and reconstruction. Based on immunity, the rat used in the experiment can be divided into two groups: The rat with normal immune capacity (e.g., Sprague-Dawley rat, Wistar rat) and nude rat without immunity. Immune suppression may occur during the repair of bone trauma. It can result in the loss of some key cell and molecular signals that regulate normal bone healing, which ultimately leads to the reduction of osteogenesis [66]. Therefore, nude mice have unique advantages in some experiments.

The most common type of rat model that uses in decellularized bone matrix and DBM is calvarial critical-size bone defect. Critical-size bone defect refers to the range of critical bone defects that cannot be spontaneously repaired at a specific bone trauma site in a given animal germline [67]. Due to the lack of muscle tissue, absent blood supply and poor bone regeneration, defect calvarial can be used to bone graft repair analysis [68]. In addition, most mammalian craniofacial and maxillofacial bones are membranous bone, and its embryos have similar origins. Therefore, the rat calvarial defect model has become an ideal model for in vivo experiments in BTE. A large number of studies have shown that the defective diameter should not more than 6 mm in the preparation of bilateral skull defects. A bone defect with a diameter of more than 7 mm can only be selected in the central region of the parietal bone [69,70]. Taking into account the number of experimental animals, costs and labor, therefore 5 mm in diameter is an ideal selection as a standard bilateral bone defect in rat calvarial.

**Figure 2:**
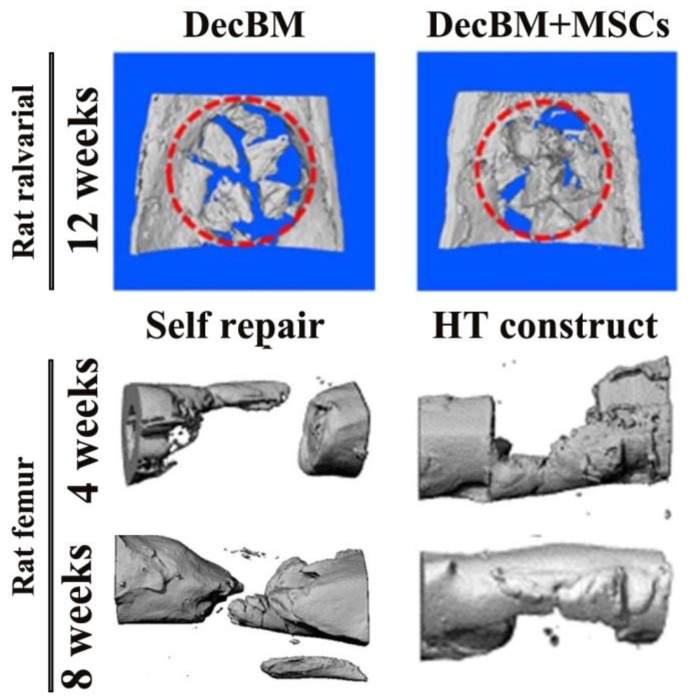
Repair and reconstruction of different natural bone grafts implanted in different sites in rat bone defect model. DecBM, decellularized bone matrix. RecBM, recellularized bone matrix. HT, hypertrophic cartilage tissues. Self repair, defect repair without any scaffold. MSCs, mesenchymal stem cells. Reproduced with permission from [58]. Copyright Lee et al., 2016. Reproduced with permission from [71]. Copyright Elsevier B.V., 2015.

In the rat calvarial defect model, it was found that decellularized bone both had excellent repair whether implanted alone or in conjunction with other bone graft materials. For example, Lee et al. [58] found good fusion of decellularized bone particles with surrounding host bone, excellent new bone formation analyzed by mineral deposit rate and histomorphology, and effective bone regeneration (Figure 2). These findings provided favorable evidence for the decellularized bone matrix as a promising bone graft. Kim et al. [59] used the decellularized bone matrix as the coating layer of the poly (lactic-co-glycolic acid) (PLGA)/poly (lactic acid) (PLA)-based mesh scaffold. After immobilizing BMP-2 and planting human placenta derived-mesenchymal stem cells (hPMSCs), they discovered that the composite scaffolds had significantly increased formation of new bone and almost complete bone defect healing.

However, there are differences among the results obtained in reconstruction of rat calvaria defect with DBM. Most studies showed that DBM, one of the components of bone implant, can significantly promote the regeneration and repair of the calvarial, such as DBM combined with PLA and stromal vascular fraction (SVF) [60], or combined with osteogenic induced ADSCs [51]. Both of them have more new bone formation and more fully filled defects than DBM alone. However, Stancoven’s [61] conclusion indicated that DBM alone significantly limits bone formation compared to other groups that incorporate with absorbable collagen sponge, recombinant human BMP-2, and exogenous parathyroid hormone. These results indicate that the use of DBM alone may not have an excellent in vivo repair although decalcification exposes abundant osteogenic growth factors (BMPs, insulin-like growth factor-I (IGF-I), etc.) and ECM proteins. Some osteogenic elements may also be absent, such as factors that promote recruitment of endogenous stem cells and promote angiogenesis.

The rat femoral defect model is also one of the extensively used defect models. Earlier studies have showed that the DBM-induced bone in rat femoral defect model had comparable energy absorption capacity and stiffness as that in the intact rat femur. The DBM-induced bone gains 35% of the normal bone’s torsional strength and its ability to deform increases under load [72]. Recently, Shi et al. [62] found that collagen-binding stromal-cell-derived factor-1α (CBD-SDF-1α) modified DBM scaffolds can effectively mobilize CD34^+^ and c-kit^+^ endogenous stem cells to the injure site after implantation for 3 days, which was favorable to the recombination of bone defect, mineralization accumulation and increase of bone mass. HE and Masson trichrome staining showed that CBD-SDF-1α modified DBM scaffolds comprised more osteoblasts and early remodeling of bone defects. Therefore, CBD-SDF-1α modified DBM scaffolds can promote bone regeneration by recruiting endogenous stem cells, which also provided more reference for designing or modifying DBM scaffolds.

In addition to the calvarial and femur defect model, numerous other rat models have also been developed to assess the in vivo defect reconstruction effects of decellularized bone matrix and DBM, such as xiphoid cartilage [63] or other osteochondral defects, spine fusion repair [64], and meniscal defect [65]. The results showed that natural bone graft can also achieve endogenous cartilage formation, repair cartilage defects of 3 mm in diameter and meniscal defects, which demonstrated the potential as “off-the-shelf” implants to promote bone tissue regeneration.

### 3.2. Rabbit Model Studies

One of the most commonly used bone defect models in rabbits is the radial defect model, which is a load-bearing site and is therefore more appropriate as a defect site than an ulnar model. This model is often used as a load-bearing model and a nonunion repair model. In addition, compared to other models of skull and middle femur defects, the modeling process of this model is relatively simple and is therefore widely developed. By establishing this defect model, our group [73] discovered that optimal new bone formation and defect healing were significantly observed in decellularized cancellous bone scaffold coated with medium-proportion of Col/HA mixture (the medium stiffness group, denoted as Col 0.5/HA 22) by assessing X-ray and micro-computed tomography (micro-CT) (Figure 2). Histological analysis of HE and Masson’s trichrome staining also confirmed that middle stiffness decellularized bone has remarkable osteogenesis, angiogenesis, Col deposition after 1 month of implantation, and significant mature bone distribution in 3 months. In a subsequent article, our group [74] also uncovered that the optimal stiffness of decellularized bone scaffolds loaded with SDF-1α have better rabbit radius repair effects. These findings demonstrate that decellularized bone graft can not only be used as an effective implant for the repair of rabbit radius, but also promote this repair after some modification (loading factor, mechanical modification etc.), and among them, the matrix stiffness-mediated signaling pathway plays a role.

**Figure 3:**
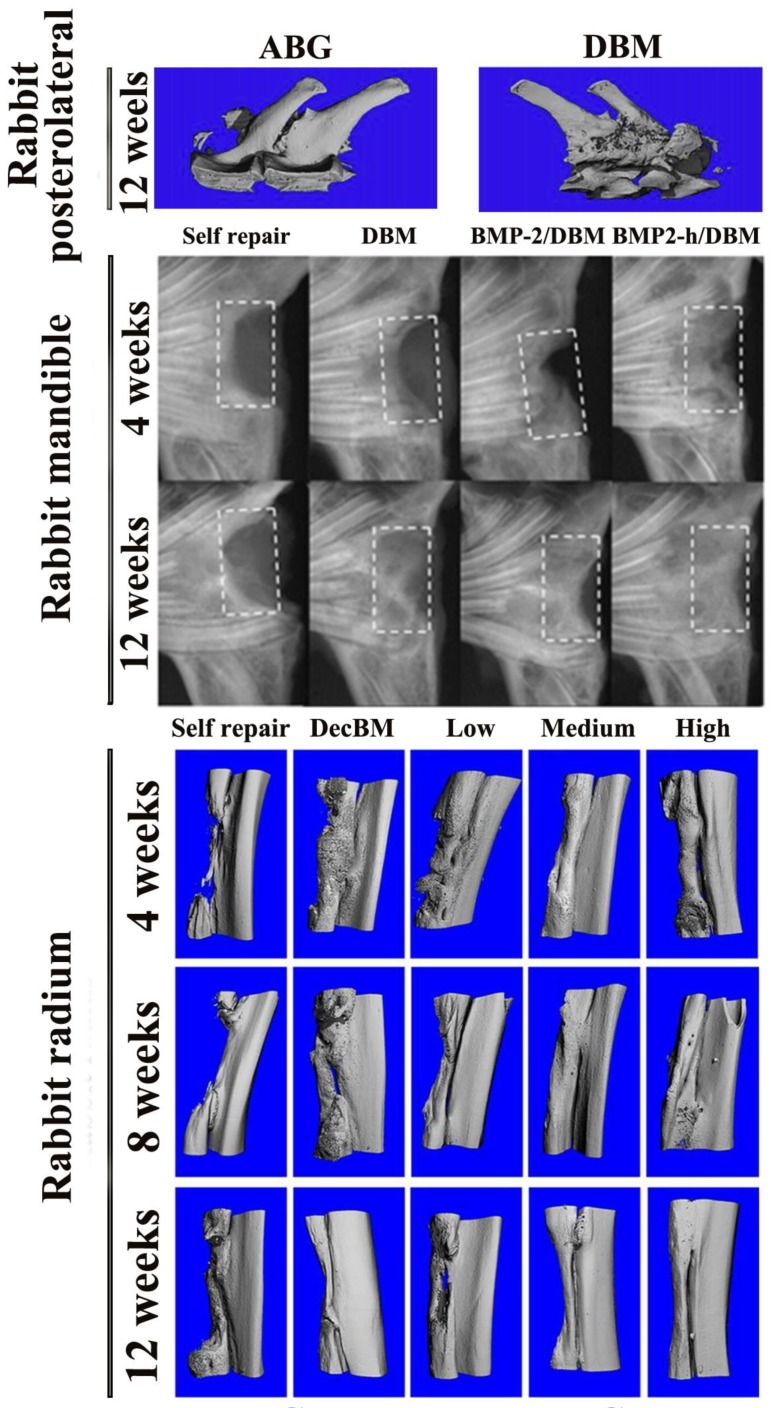
Repair and reconstruction of different natural bone grafts implanted in different sites in rabbit bone defect model. DecBM, decellularized bone matrix. Self repair, defect repair without any scaffold. Low, low stiffness decellularized bone composite scaffold. Medium, medium stiffness decellularized bone composite scaffold. High, high stiffness decellularized bone composite scaffold. DBM, demineralized bone matrix. BMP2-h, collagen-binding bone morphogenetic protein-2. ABG, autologous bone graft. Reproduced with permission [73]. Copyright John Wiley & Sons, 2016. Reproduced with permission [42]. Copyright Elsevier B.V., 2007. Reproduced with permission [83]. Copyright Elsevier B.V., 2014.

Still, other rabbit models such as femur [75], tibiofibular [76], ulna [77,78], cranial [79,80], mandible [42,81], humeral [82] posterolateral [83] (Figure 3) and osteochondral defect models [84,85] are occasionally used to assess the in vivo restoration of natural bone graft.

As mentioned earlier, decellularized bone matrix and DBM are decreasingly being investigated as separate bone graft because it can bring some inflammation and foreign body reaction [81]. Therefore, in various parts of rabbit defects, these natural bone grafts are often combined with stem cells that have multidirectional potential and satisfactory proliferative potential, such as ADSCs and bone marrow-derived mesenchymal stem cells (BMSCs), or combined with contributing bone-related factors, proteins or functional substances to be researched together in vitro and in vivo.

In the first category, the stem cells can be anchored as seeding cells on a suitable scaffold to successfully repair the defect in the autologous environment. Previous studies showed that the osteogenesis of MSCs could be promoted by modifying the expression of cytotoxic T lymphocyte-associated antigen 4 (CTLA4). However, the mechanism is not clear. Zhang et al. [86] investigated the bone repair of DBM combined with MSCs that was modified to express CTLA4 in a rabbit radius defect model. It was confirmed that modified MSCs combined with DBM repaired better than unmodified MSCs or DBM alone, and this better repair is associated with up-regulation of Wnt/β-catenin signaling. Kaempfen et al. [82] implanted the decellularized bone matrix seeded with bone marrow MSCs either directly in situ or implanted immediately or only “subcutaneously” for 6 weeks after combining with blood vessels. Bone formation and remodeling of the graft was assessed by tartrate resistant acid phosphatase (TRAP) detection of positive osteoclasts. At the same time, quantitative examination of necrotic tissue was performed. It was found that the two-step strategy after incubation resulted in significantly more blood vessels than the one-step strategy, but the necrotic area had no significant difference between them. These results indicate the development of suitable engineered bone graft still requires better pre-vascularization, better absorbability and higher osteogenesis.

In the second category, transforming growth factor-β1 (TGF-β1) [79], Oncostatin-M (OSM) [80], B2A2-K-NS [78] and CBD-BMP-2 [42] were separately loaded into the DBM in different forms. Then, the ability to induce new bone formation in rabbit ulna, cranial, ulna, and mandibular defect models was examined. The results showed that the dose of delivery factor correlates with the osteoinduction capacity. In rabbit ulnar defect models, osteoinductive was enhanced with an increased TGF-β1 dosage [79]. OSM, a member of the interleukin-6 (IL-6) cytokine family, plays a role in maintaining bone homeostasis and supporting bone regulation. However, in the rabbit cranial defect model, the new bone area is inversely proportional to its dose [80]. B2A2-K-NS, which can significantly promote BMP-2-induced alkaline phosphatase (ALP) activity and mineralization, also augments the osteoinductive environment in the rabbit ulnar defect model, which manifest in enhanced mineralization and higher bone mineral density value [78]. Compared to BMP-2 lacking CBD, combining CBD and the N-terminus of native BMP-2 was able to specifically bind collagen and maintain a more complete biological activity. The implantation of scaffolds loaded with CBD-BMP-2 proved significant osteoinductive properties and excellent homogeneous bone formation in rabbit mandibular defect model [42] (Figure 3). Both of these results indicate that seeding cells or functional molecules induce better bone formation not only in quantity but also in quality, after specifically loaded or bound to the target bone repair system.

In addition, the in vivo bioreactor principle has been considered as a promising method to reconstruct bone defects. In detail, it uses the body as a living bioreactor to culture stem cells, biological scaffolds and growth factors, and uses the body’s self-regenerating ability to regenerate new tissue. Periosteal has the ability to significantly induce bone growth and remodeling, indicating its suitability as an in vivo bioreactor strategy for prefabricating bone grafts [87]. For example, Huang et al. [87], combined a pedicled periosteal flap with decellularized bone matrix to prefabricate vascularized functional bone graft in a rabbit model. Bone tissue was induced on the surface of decellularized bone matrix detectived by micro-CT at 8 and 16 weeks postoperatively. Biomechanics and histomorphometry studies indicated that the periosteal graft group showed a larger bone mass, faster bone formation rate, higher vascular density and stronger biomechanical properties.

### 3.3. Other Animal Model Studies

**Figure 4:**
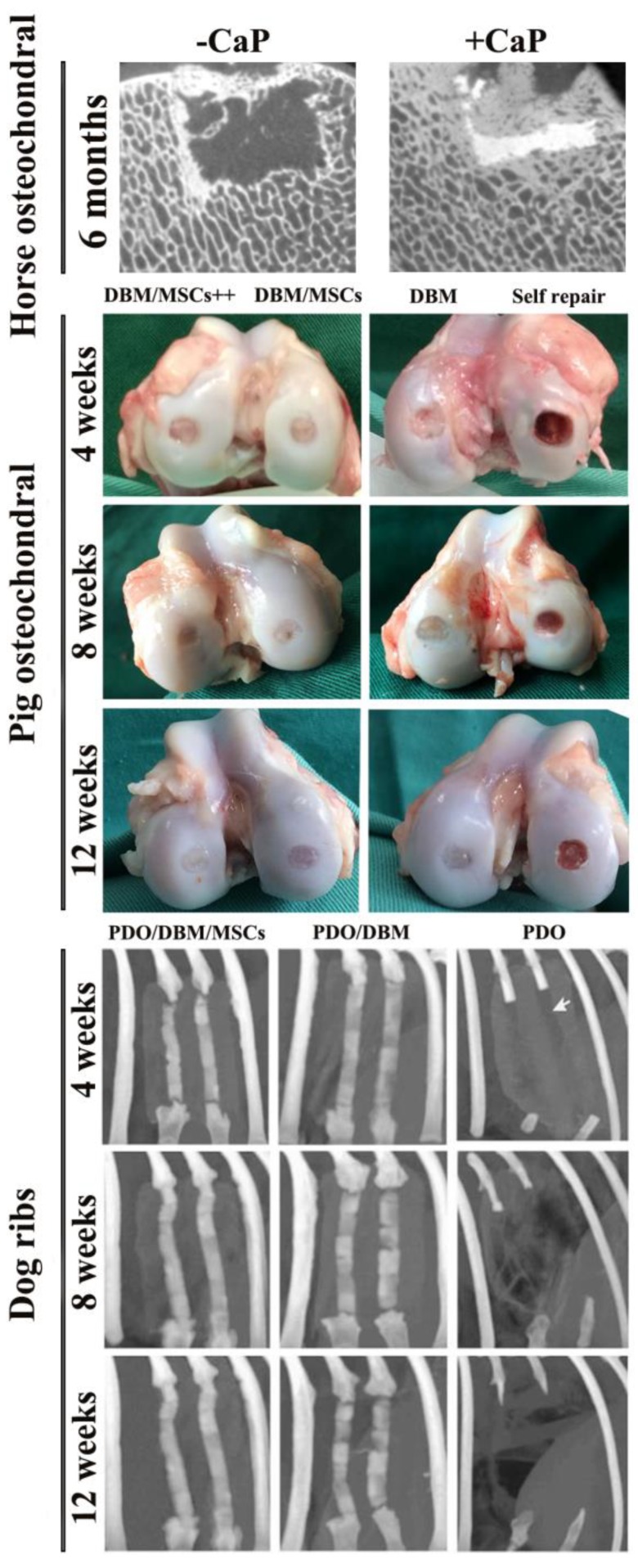
Repair and reconstruction of different natural bone grafts implanted in different sites in the rabbit bone defect model. Self repair, defect repair without any scaffold. MSCs, mesenchymal stem cells. −CaP, decellularized bone scaffold without calcium phosphate. +CaP, decellularized bone scaffold with calcium phosphate. DBM, demineralized bone matrix. PDO, polydioxanone. DBM/MSCs++, a combination of DBM and MSCs infected with adenovirus-mediated-bone morphogenetic protein (Ad-BMP-2) and transforming growth factor-β3 (Ad-TGF-β3). Reproduced with permission from [92]. Copyright Elsevier Inc, 2017. Reproduced with permission from [41]. Copyright Wang et al., 2014. Reproduced with permission from [93]. Copyright Elsevier B.V., 2009.

In addition to the most common mouse and rabbit models, some of the other larger animals, such as pigs [41,88] (Figure 4), dogs [89,90] sheep [91], and horses [92] (Figure 4) are also applied to assess the ability to reconstitute different defect sites with natural bone graft. A number of other factors have also been investigated in these studies, including the role of calcium phosphate, and the addition of polydioxanone and cytokines (TGF-β3, BMP-2) (Figure 4). It is obviously visible that different experimental groups have different repair results.

The complete repair of osteochondral defects has been a major obstacle in the field of BTE because of the complex three-layered structure of cartilage (hyaline cartilage, calcified cartilage and subchondral bone). Currently, the maximum frequency used animal models for repairing this defect are small animal models, while the experimental data provided by large animal models is still relatively limited. The results of the study by Vindas Bolaños [92] suggest that small animal models may overestimate the true in vivo repair effect of implants and it is necessary to construct large animal defect models and with a long-term study for assessing the healing of implants. In detail, horse cartilage-derived decellularized scaffolds were combined with calcium phosphate and implanted into the cartilage defect site of the medial trochlear ridge of the horse femur. The defect was incompletely filled at 2 months. At 6 months, although well-integrated, there was no significant difference between groups with and without calcium phosphate, and the high fibrosis tissue repair, high expression of type I Col, and low expression of type II Col all indicated the poor repair in this large animal model (Figure 4). However, a 4.2 mm (diameter) × 6 mm (depth) high articular load bearing canine cartilage defect model (femoral condyle) was constructed in Yang’s study [89]. The ECM-derived decellularized scaffold/cartilage-induced bone marrow MSCs constructs were found to have a higher histological score than the scaffolds without cells, and cartilage hardness (6.95 ± 0.79) N/mm in the experimental group (6 months) was 70.77% of normal cartilage. Osteochondral bone strength of the experimental group was (158.16 ± 24.30) N/mm, which was 74.95% of normal tissue. Mature trabecular bone was regularly formed at 3 months and 6 months. The results demonstrated that decellularized matrix as one of the composite components of bone implants can present the potential to repair large, highly loaded osteochondral defects.

In addition, large animal models are commonly used for the reconstruction of facial bones. Reconstruction of facial bone poses great challenge because of the need to accurately reconstruct the original appearance and functional organization, which requires the design of the most complex facial bone graft. The current standard method is to collect bone from other parts of the body to reconstruct the defect site, but this inevitably brings massive limitations, including surgery-related pain and complications. Bhumiratana et al. [88] used clinically approved decellularized calf trabecular bone as an implant, which was processed into anatomically correct shapes by image-guided microfabrication. Then, the scaffold loaded with autologous adipose derived stromal/stem cells was perfused in specialized bioreactor for 3 weeks to form mature bone tissue. After implanting into a bone-mature Yucatan mini pig (a preclinical human model), the implanted decellularized bovine bone combined well with native tissue after 6 months later, resulting in more new bone and greater vascular penetration than the self repair group. This study demonstrated that natural bone can also be designed into a custom shape, and has great potential for accurate repair of personalized orthopedic and cranial and maxillofacial bones. In a sheep vertebral bone void model, Fujishiro et al. [94] have compared the efficacy of a new bone graft substitute composed of a combination of mineralized and demineralized allograft, along with hyaluronic acid (AFT Bone Void Filler) with several other bone graft materials. The results of histology and quantitative histomorphometry demonstrated that all of the bone defects in the AFT DBM preparation group showed good new bone formation with variable amounts of residual DBM and mineralized bone graft. This study illustrated the potential of a mixture of mineralized and demineralized allograft as an alternative to autologous transplantation.

Of course, a few evaluation experiments were conducted on the human body. This part of the study is even more persuasive. After all, the animal model was originally designed to simulate the final human experiment. Decellularized bovine trabecular bone loaded with autologous MSCs was implanted in bearing large segmental long bone defect (distal 72 mm defect of the tibia and fixation with intramedullary nail) of a 58-year-old female after perfusion culture [95]. New bone formation was observed, and patients were free to walk after 6 weeks. Additionally, one male and three female patients with primary mandibular tumors underwent a half ear resection [96]. After resection of the tumor, three cases of mandibular defects were reconstructed with an autologous rib graft. One case was implanted with 3D decellularized cancellous bone contained with paracrine factor from bone marrow MSCs. Preliminary clinical study showed that decellularized bone contained with paracrine factor can be used to reconstruct large mandibular defects after tumor resection. These clinical results indicated that decellularized bone matrix as bone graft in clinical application does have an excellent reconstruction effect.

## 4. Discussion

Numerous studies showed that the ability of scaffolds to repair and regenerate depends on many factors, including composition, fabrication method, macroscopic and microscopic structural, mechanical characteristics, premodification and whether coated with growth factors. By analyzing the composition of decellularized bone and DBM, we concluded that the bone microenvironment of the natural bone matrix is retained after these two treatments. Simultaneously, the 3D microporous structure is conducive to the adhesion and spreading of endogenous stromal cells and collagen deposition of ECM. In addition, the decellularization removes the immunogenicity of the implants, and the extracellular microenvironment constitutes a complex network that connects the tissue structure, which plays a key regulatory role in cell physiological activities such as cell survival and development. The natural ECM (divided into Col, glycoprotein, aminoglycan and proteoglycan, elastin four categories) has good mechanical properties, cell compatibility and biodegradability [97,98]. As for DBM, decalcification revealed more bone-related factors and proteins. In order to better understand the in vivo restoration of these two functionalized natural bone grafts and their possible adverse effects, further investigation and evidence collection are still required before clinical trials.

In vivo bone regeneration and remodeling of decellularized bone matrix and DBM in different animal species such as mice, rabbits, dogs, pigs, sheep, as well as different defect sites such as the femur, tibia, mandible, radius and calvarial of the bone, were reviewed in the present study. Heterologous decellularized bone matrix can be designed as a personalized implant with complex custom geometry, and provide feasibility for personalized BTE especially the facial bone reconstruction [88]. The results of a large animal defect model indicate that decellularized bone matrix can demonstrate the potential for repairing large, high load-bearing osteochondral defects [89]. In addition, after some modifications (loading cells [82], cell factors [74], mechanical modification [73]), this repair can be improved. However, studies have proved that decellularized bone that is not compounded with other materials, cells, or factors does not form significant new bone and vascular remodeling, whereas a foreign body response characterized by fibrocapsule formation was observed [81]. This also suggested that there remains demand for the development of suitable engineered bone grafts with better absorbability and higher osteogenesis, better pre-vascularized treatment [82].

Isolated DBM showed limited bone formation [61]. Analysis also showed that there may also be a lack of certain osteogenic elements, especially the cell factor that improves endogenous stem cell recruitment and promotes angiogenesis. For example, vascular endothelial growth factor (VEGF)-loaded DBM scaffold improves vascularization of the scaffold [98]. CBD-SDF-1α-modified DBM scaffolds can efficiently mobilize endogenous stem cells and home to the injured areas, which is beneficial to the reconstruction and healing of femur defect in rats [62]. In addition, DBM is often combined with other bone graft materials [60] and stem cells (e.g., ADSCs [51,77] and MSCs [86]) to investigate the in vivo activity of each group of grafts. The study found that DBM composite scaffolds have increased new bone formation and almost complete bone defect healing in vivo, demonstrating the potential of natural bone implants to promote tissue regeneration. These findings provide experimental guidance for understanding the choice of animal models, in vivo repair and further functional modifications of natural bone composites.

We acquired the following considerations. First, the choice of natural bone scaffolds needs to be based on the type of bone to be restored (cancellous bone, dense bone, or cartilage) when considering defect repair for a particular animal species. If necessary, further modifications (stiffness as much as possible to match the host bone, loading cells, coating growth factors or immune regulatory factors) may be made. The superior repair and regeneration capabilities of grafts are also related to the manufacturing process of material. A large number of available 3D printing techniques and materials confirm that some of them may be feasible, but further research is still needed. In the 3D manufacturing process, additive manufacturing (AM) can bring us more and more surprises, such as 3D scanning, design and analysis opportunities. In addition, AM has the potential to improve performance and efficiency, improve field operation and save costs.

Secondly, conversely, when evaluating the bone repair and reconstruction of the prepared bone graft, it is necessary to select suitable animal defect sites. For example, due to lack of muscle tissue, poor blood supply and poor bone regeneration, the cranial defect model became the most commonly used non-load-bearing bone defects in BTE [68]. The femoral, mandible, and articular cartilage on the surface of the femoral condyle is an ideal candidate for high load-bearing bone defect model, craniofacial and maxillofacial defect model and cartilage defect model, respectively.

Taken together, these two kinds of natural bone materials have massive advantages as bone grafts in BTE, but the limitations should also be taken into consideration at the same time. In the actual design, through loading cells, coating specific functional factors and other methods, the function of decellularized bone matrix and DBM as the main framework of the matrix can be developed more profitably.

## 5. Conclusions

The distinctive capability of natural bone implants to repair and reconstruct not only depends on the specific compositional structure, especially the retained natural bone microenvironment (macrostructural, microstructural, mechanical, etc.), but also on the manufacturing methods of decellularized bone and DBM, such as decellularization (physical, chemical and enzymatic methods) and the choice of decalcification reagents (hydrochloric acid and EDTA-2Na, etc.), which greatly affect the biological performance of the prepared scaffolds. Additionally, pre-modified decellularized bone and DBM, such as surface physical modification, surface chemical modification, modification of matrix stiffness, loaded with seeding cells and coated with growth factors or other functional molecules prior to implantation, also have been demonstrated to have a significant effect on bone healing and reconstruction. These modifications are necessary to further fabricate more functional bone grafts, and these modifications can have a consequential impact on in vivo results. Therefore, in order to better understand the involved exact mechanisms and adverse effects, further investigation is required by animal experiments prior to clinical trials. Animal species, defect size and implantation time period will affect in vivo healing and repair of implanted grafts. Due to small size and characteristics of convenience processing, rats and rabbits are the most commonly used animal models. However, for their poor similarity to human bones, further investigations and more research on large animal models that are close to human skeletal characteristics is still necessary. Taken together, based on the information provided in this overview, it was confirmed that decellularized and DBM in the natural bone grafts can in fact reconstruct defective bone, even though the effects of different pretreatments still require further investigation in clinically relevant animal models of bone defects. By analyzing some of the existing research results, the authors hope that this review will provide a reference for the in vivo studies and new design of decellularized and DBM scaffolds to obtain preferable in vivo regeneration.

## Figures and Tables

**Table 1:** Comparison of decellularized and DBM scaffolds.

Comparison Category	Decellularized Bone Matrix Scaffold	DBM Scaffold
Preparation method	Physical methods (snap freezing, mechanical force and mechanical agitation, etc.) [25]	Treated with decalcification reagents (hydrochloric acid and EDTA-2Na, etc.)
	Chemistry methods (alkaline solution, acid, nonionic detergents and Tritonn X-100, etc.) [26]	
	Enzymatic methods (exonucleases, endonucleases and trypsin, etc.) [27]	
Characteristics	Effectively remove cells from host bone tissue	A complex consisting of collagen, non-collagen, and lower concentrations of growth factors
Advantages	Reduce or eliminate the antigenicity of bone	Decalcification exposes osteogenic factors
	Suitable mechanical strength; better biocompatibility	Good biological properties, osteoinduction and bone conduction activity
	The same structure and composition as natural bone	Biodegradable
		Weak immunogenicity
		Maintain natural bone-like pore structure and 3D structure
Disadvantages	Decellularization still causes damage to natural ECM components and microstructures	Low biomechanical strength
	The difference in ECM from different donor sources is difficult to exclude	Not suitable for repairing bone defects in load-bearing defect models
	It is difficult to completely avoid inflammation and immune response	

**Table 2:** The in vivo results of different natural bone grafts implanted in different sites in rat bone defect model.

Defect Sites	Grafts	Descriptions	References
Calvarial	Decellularized bone	Well new bone fusion around the particles	[58]
Calvarial	Decellularized bone matrix as the coating layer of the poly PLGA/PLA scaffold	Increased formation of new bone	[59]
Calvarial	DBM combined with PLA and SVF	Significantly promote the regenerative repair	[60]
Calvarial	DBM combined with ADSCs	Have more new bone formation	[51]
Calvarial	DBM	Significantly limits bone formation	[61]
Femur	CBD-SDF-1α modified DBM scaffold	Effectively mobilize CD34+ and c-kit+ endogenous stem cells to the injure site	[62]
Xiphoid cartilage	Decellularized human bone matrix scaffold	Achieve endogenous cartilage formation	[63]
Spine	DBM fibers	Promoted spine fusion repair	[64]
Meniscal	Decellularized meniscus ECM hydrogel	Contribute to tissue regeneration and protection from joint space narrowing	[65]
